# Accuracy of Machine Learning to Predict Upper-Limb Outcome Within the First 72 Hours Poststroke

**DOI:** 10.1161/STROKEAHA.125.054989

**Published:** 2026-06-10

**Authors:** Govert J. van der Gun, Ruud W. Selles, Carel G.M. Meskers, Erwin E.H. van Wegen, Gert Kwakkel, for the ICAI Stroke Lab, Daniël Bos

**Affiliations:** 1 Department of Rehabilitation Medicine (G.J.v.d.G., R.W.S.), Erasmus MC, University Medical Center Rotterdam, the Netherlands.; 2 Department of Plastic, Reconstructive, and Hand Surgery (R.W.S.), Erasmus MC, University Medical Center Rotterdam, the Netherlands.; 3 Department of Rehabilitation Medicine, Amsterdam UMC, Vrije Universiteit Amsterdam, the Netherlands (C.G.M.M., E.E.H.v.W., G.K.).; 4 Amsterdam Movement Science and Amsterdam Neuroscience Campus, the Netherlands (C.G.M.M., E.E.H.v.W., G.K.).

**Keywords:** decision support techniques, feasibility studies, ischemic stroke, machine learning, recovery of function, upper extremity

## Abstract

**BACKGROUND:**

Timely and accurate prediction of poststroke motor outcome is important for efficient rehabilitation planning and resource allocation. Existing bedside models for predicting upper-limb outcome after stroke require further refinement to be effectively implemented in stroke units within the first 72 hours. This study aimed to develop and internally validate a machine learning model to predict the 6-month Action Research Arm Test score using simple clinical tests commonly assessed within the first 3 days poststroke.

**METHODS:**

In 296 first-ever ischemic stroke patients pooled from 4 prospective Dutch cohort studies across 44 centers (2000–2019), we compared the cross-validated prediction performance of multiple eXtreme Gradient Boosting models using different sets of bedside clinical tests to predict the 6-month Action Research Arm Test outcome (0–57). We then selected the model with the minimal predictor set that best balanced bedside feasibility and accuracy and validated it within 72 hours poststroke on a test data set (n=32) from the same cohort using median absolute error as the evaluation metric.

**RESULTS:**

A model incorporating Shoulder Abduction from the Motricity Index, voluntary finger extension, Fugl-Meyer Upper Extremity, and total National Institutes of Health Stroke Scale score as bedside tests, showed the best tradeoff between model simplicity and predictive accuracy (median absolute error, 5.9 on the 0–57 score; interquartile range, 2.9–12.9).

**CONCLUSIONS:**

Our model predicts the 6-month Action Research Arm Test score using a minimal set of bedside clinical tests collected within the first 3 days after stroke, achieving a median absolute error below the Action Research Arm Test minimal clinically important difference of 6 points.

An estimated 70% to 80% of stroke survivors experience upper-limb impairment immediately after onset, and 30% to 60% continue to have deficits at 3 to 6 months.^1,2^ Longitudinal studies indicate that upper-limb recovery results from both spontaneous neurobiological restitution and learning-dependent substitution.^3–5^ These studies suggest that spontaneous behavioral restitution plateaus within the first 8 to 10 weeks after onset, after which further improvements are likely adaptive.^6^ Serially measured kinematic data indicate that this period coincides with the return of movement quality, including intralimb coordination^7^ and smoothness of multijoint movements.^8^ Because this early phase reflects neurobiological recovery, predicting recovery potential should begin as early as possible to support discharge planning and avoid unnecessary transitions between care settings.^9–11^

Despite the availability of multiple models to predict upper-limb outcome after stroke,^12^ their applicability in acute stroke units is limited. A key limitation is that most models are validated beyond the first 72 hours, whereas clinical decisions in stroke units occur within this window. To our knowledge, only 3 recent models have been evaluated within 72 hours.^13–15^ This likely reflects the absence of inception cohorts in which subjects are recruited at the point of admission to an acute stroke unit.^16^ Moreover, many prediction models rely on linear regression and fail to capture the nonlinearity and complex individual variation in recovery.^12^ Finally, several prognostic studies have investigated the predictive value of neuroimaging, such as Diffusion Tensor Imaging,^17^ and neurophysiological measures, such as Transcranial Magnetic Stimulation-evoked motor potentials, alongside clinical tests.^13,18^ However, these models may not be practical because their implementation in routine care is limited by time, costs, and staffing constraints. Therefore, optimizing patient-specific prediction accuracy using simple, recommended clinical bedside tests such as the Shoulder Abduction item from the Motricity Index,^14,15^ Voluntary Finger Extension assessed with the Fugl-Meyer Assessment,^14^ the Fugl-Meyer Upper Extremity (FM-UE) total score,^2,19^ and items from the National Institutes of Health Stroke Scale (NIHSS)^16^ is likely the most practical approach to improve clinical decision-making within the first days poststroke.^1,15,20^

The present study investigates whether machine learning models such as eXtreme Gradient Boosting (XGBoost), based on recommended clinical bedside tests measured within 72 hours after stroke, can generate accurate patient-specific predictions of upper-limb capacity at 6 months. We build on earlier work that used similar methods but relied on tests impractical at the bedside.^21^ Here, we develop and internally validate an XGBoost model to predict the 6-month Action Research Arm Test (ARAT) score, a widely used and well-validated measure of upper-limb capacity that is responsive to clinically meaningful change after stroke. To preserve clinically relevant information, ARAT was predicted on a continuous scale with a target prediction error below the Minimal Clinically Important Difference of 6 points.^22^

## Methods

The study population and methodology followed Van der Gun et al^21^ and are summarized here. The data that support the findings of this study are available from the corresponding author upon reasonable request.

### Study Population

We combined prospectively collected data from 4 Dutch cohort studies: EPOS (Early Prediction of Functional Outcome After Stroke),^14^ EXPLICIT (Explaining Plasticity After Stroke),^23^ 4D-EEG,^24^ and EXPLORE. These included first-ever ischemic stroke survivors recruited between 2000 and 2019 from 44 collaborating centers across 4 regional university districts. Data comprised demographics, stroke characteristics, ARAT scores, and other clinically recommended assessments of the most affected upper limb,^19^ along with measurement times in days poststroke. At each visit, all clinical measures were obtained within a single measurement session. We included patients with at least 2 serial measurements, with the final measurement taken at 180±14 days poststroke. No other criteria were applied. Baseline characteristics of included and excluded patients were compared using absolute standardized mean differences to assess representativeness of the included sample. Baseline was defined as the first available measurement.

### Model Development

We developed an XGBoost model, a tree-based machine learning method that builds an ensemble of sequential decision trees,^25^ to predict patient-specific upper-extremity capacity at 6 months as measured by the ARAT. We randomly split the data set at the patient level into 2 balanced subsets: a training set (n=237, 80%) and a test set (n=59, 20%). The optimal model configuration was selected using 5-fold, 5-repeat cross-validation within the training set. Categorical predictors were encoded as binary values for each level. The final model was a bootstrap ensemble of XGBoost models (Figure S1). Prediction intervals were derived from the bootstrap prediction errors.

### Predictor Selection

We took a pragmatic approach to predictor selection, balancing prediction performance with bedside assessment feasibility. As a base set, we included Shoulder Abduction from the Motricity Index and Voluntary Finger Extension, both of which have been shown to be effective predictors of upper-limb outcome after stroke.^13,14^ Building on this, we added additional clinical measures as potential predictors that capture different aspects of the stroke recovery process and are suitable for bedside assessment. These included overall upper extremity function (FM-UE), stroke severity (NIHSS), motor impairment (MI-arm and MI-leg), sensory deficits (NIHSS Sensory, item 8), stroke type (Bamford Scale), and various combinations of these measures. Baseline ARAT was excluded because its equipment (eg, ARAT box, table, and chair) may be impractical in acute stroke units. For each combination, we evaluated the model’s cross-validated median absolute error (MedAE) among patients in the training set with measurements within the first 72 hours poststroke (n=129/237, 54%). We selected the set that yielded the best performance by visually comparing box plots of the prediction error distributions while ensuring the practical feasibility of bedside measurement during the acute phase. Practical feasibility was defined as minimizing the number of predictors while maintaining performance and prioritizing routinely collected measures. The final model was trained on all patients in the training set.

### Model Evaluation

Model performance was evaluated in test-set patients measured within 72 hours poststroke (n=32/59, 54%) using the absolute error between predicted and observed 6-month ARAT scores. Error distributions were visualized using box plots.

All analyses were conducted in R (version 4.3.1). The full source code (https://gitlab.com/icai-stroke-lab/xgboost-arat-bedside) and an online tool (https://icaistrokelab.shinyapps.io/XGBoost-Bedside-ARAT-Predictions) are available for testing and validating the model in patient cohorts. This study is reported in accordance with the TRIPOD-AI guidelines (Transparent Reporting of a Multivariable Prediction Model for Individual Prognosis or Diagnosis - Artificial Intelligence).

## Results

### Study Population

Of the 451 patients from the 4 cohort studies (Table), we included 296 who had at least 2 measurements, with the final measurement taken within ±14 days of the 6-month mark. Among the included patients, the baseline measurement was taken at a median of 3 days poststroke (interquartile range [IQR], 2–7; Figure S2). Of these, 54% (n=161/296) had their baseline measurement taken within the first 72 hours of stroke onset (median, 2 days; IQR, 1–3). Of all included patients, 40% (n=118/296) had a baseline FM-UE score of 10 or lower, reflecting a strongly right-skewed distribution. The mean ARAT score at 6 months was 34 (SD, 24). No data were missing for any predictor or outcome variable. Baseline characteristics were comparable between included and excluded patients (Table S1).

**Table.**
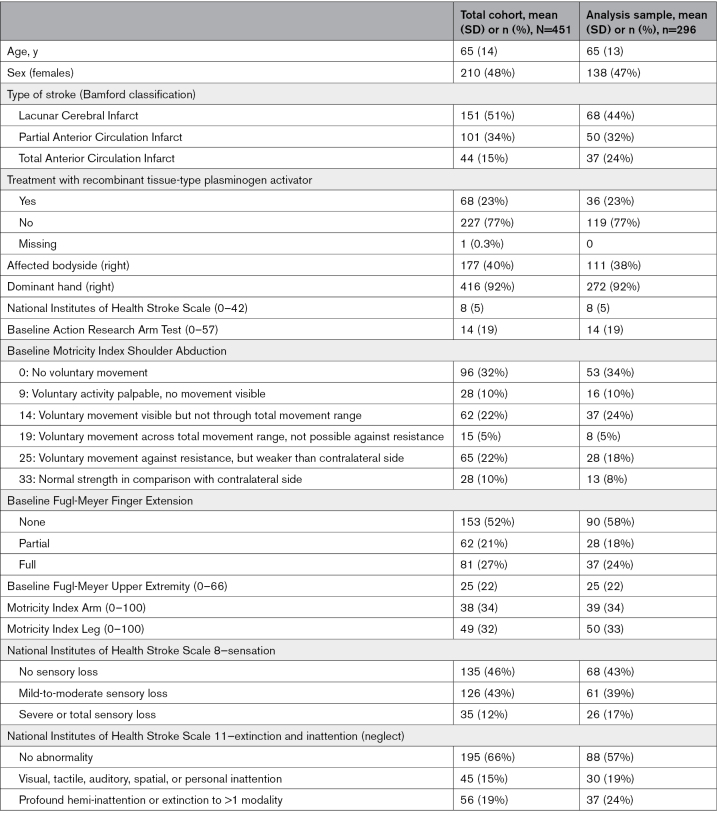
Patient Characteristics at Baseline (First Available Measurement: Mean=5 Days, SD=4.6 in Total Cohort) for the Total Pooled Cohort and the Analysis Sample

### Predictor Selection

**Figure 1.**
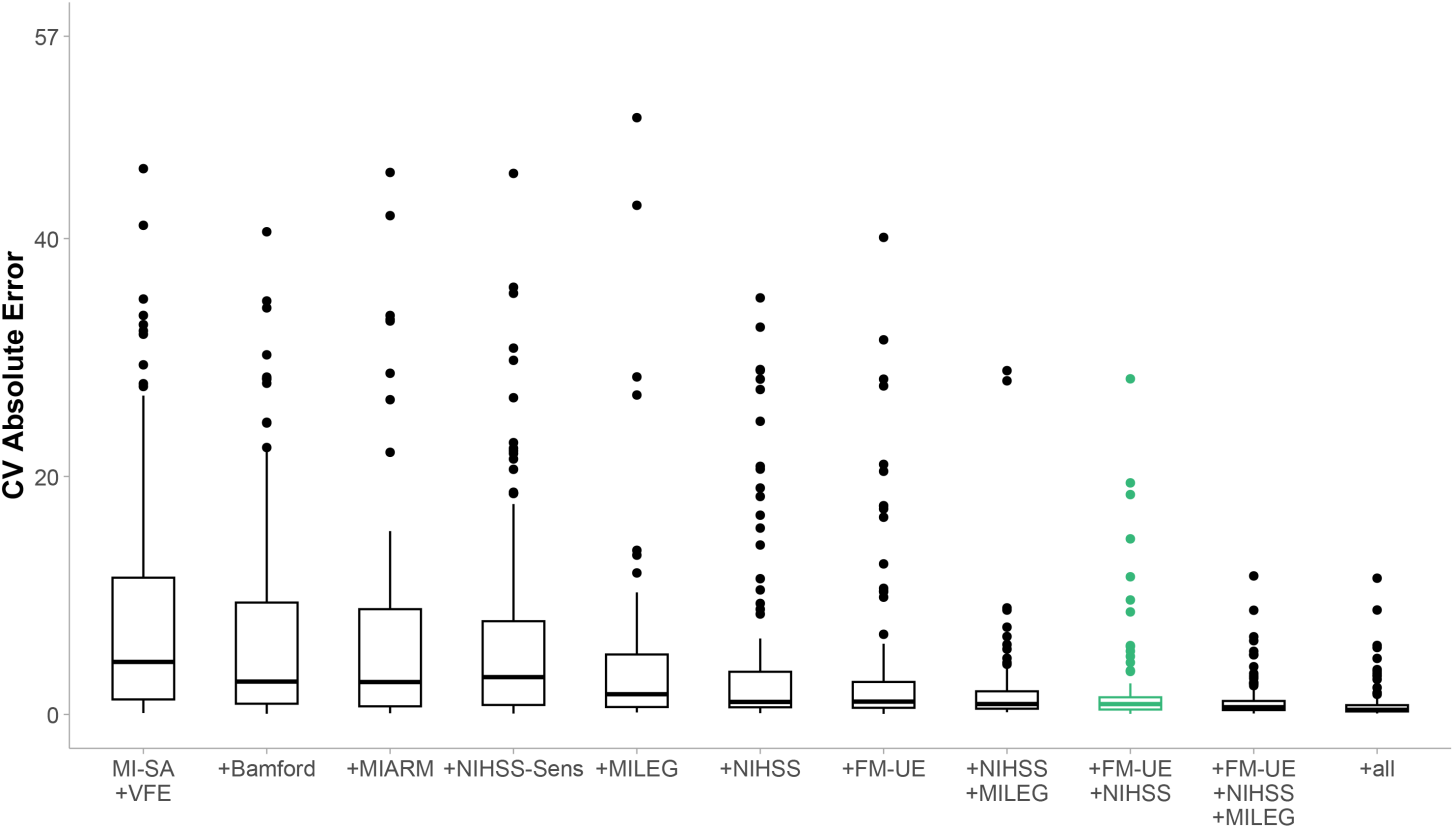
**Box plots of cross-validated (CV) model performance for predicting the 6-month Action Research Arm Test score at 72 hours poststroke (n=129) using different feature sets.** The box plots display the median, interquartile range (IQR), lower whisker (Q1−1.5×IQR), and upper whisker (Q3+1.5×IQR), with outliers represented as dots outside this range. The combination of the base model (Shoulder Abduction from the Motricity Index [MI-SA] + Voluntary Finger Extension [VFE]), Fugl-Meyer Upper Extremity (FM-UE), and National Institutes of Health Stroke Scale (NIHSS; highlighted in green) was selected as the optimal model for routine clinical use. MI-ARM indicates Motricity Index of the arm; and MI-LEG, Motricity Index of the leg.

Cross-validated absolute error distributions of patients from the training set who had a measurement within the first 72 hours poststroke (n=129/237, 44%) across different predictor sets are shown in Figure 1. The model incorporating all clinical measures demonstrated the best performance in the training data, with the lowest average error and error dispersion (MedAE=0.4; IQR, 0.3–0.8). However, this comprehensive set may be impractical for routine clinical use. We therefore selected a simplified model based on FM-UE, NIHSS, Shoulder Abduction from the Motricity Index, and Voluntary Finger Extension. This model achieved comparable performance (MedAE=0.9; IQR, 0.4–1.4) in the training and was selected for further evaluation on the test set.

### Model Evaluation

**Figure 2.**
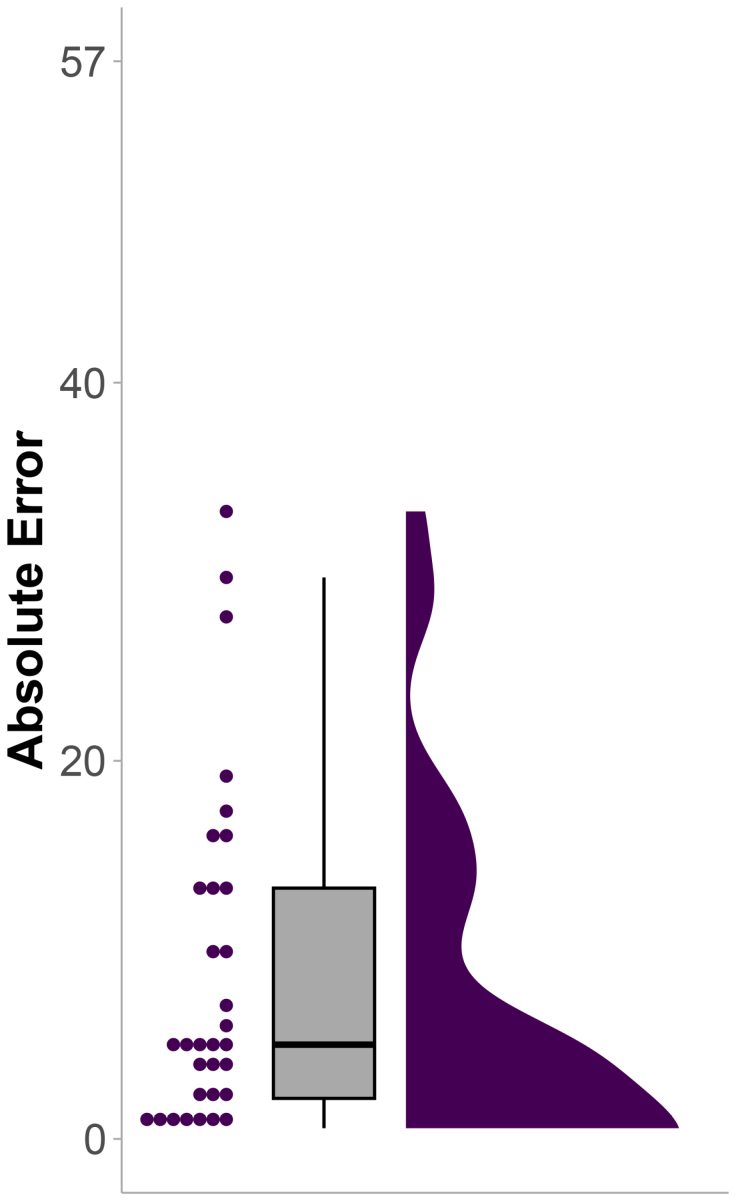
**Six-month Action Research Arm Test prediction errors for the model in 32 patients from the test set who had a measurement within 72 hours poststroke.** Box plots depict the median, interquartile range (IQR), lower whisker (Q1−1.5×IQR), and upper whisker (Q3+1.5×IQR), with outliers indicated as points outside this range. Individual prediction errors are represented as dots, and the lateral density plot illustrates the distribution of these errors.

Of the 59 test-set patients, 32 (54%) had a baseline measurement within 72 hours poststroke. Applied at this timepoint, the model including Shoulder Abduction from the Motricity Index, Voluntary Finger Extension, FM-UE, and NIHSS achieved a MedAE of 5.9 points (IQR, 2.9–12.9) for predicting the 6-month ARAT total score. At 24 hours poststroke, the MedAE was 6.2 points (IQR, 4.3–14.0) among the 10 patients from the test set with available data at this time point. These errors exceeded those in Figure 1 because Figure 2 reflects performance in held-out patients, providing a more realistic estimate of real-world performance.

Three examples of individual predictions are shown in Figure S3.

## Discussion

We developed and validated an XGBoost-based machine learning model that enables accurate predictions of 6-month upper-limb capacity (ARAT total score) using simple, bedside clinical tests assessed within the first 3 days poststroke. The model achieved a median prediction error of 5.9 points (IQR, 2.9–12.9), which approximates the 6-point Minimal Clinically Important Difference of the ARAT at 6 months.^22^ It provides patient-specific ARAT predictions and prediction intervals that quantify individual uncertainty. Because predictors were selected through cross-validation and the model was evaluated on a test set, we expect our model to generalize reasonably well to new cohorts.

Although the current model yields accurate predictions for most patients, performance is reduced in those with severe early upper-limb paresis, defined here as FM-UE below 18 in the first 3 days. In this subgroup, recovery trajectories diverge markedly: some patients (n=5/59, 8% of the test set), referred to here as super-recoverers, show rapid and excellent recovery, while most low-baseline starters exhibit little to no recovery. This heterogeneity has been reported previously and is consistent with our earlier work.^2,26^ Point predictions in this subgroup should therefore be interpreted with caution, and individual prediction intervals, which are wider for these patients, indicate where predictions are less reliable. Figure S4 illustrates prediction performance stratified by 3 baseline FM-UE severity groups.

Accurately distinguishing super-recoverers from other patients with severe impairment likely requires information beyond early clinical motor scores alone. Neurophysiological markers of corticospinal integrity, such as motor-evoked potentials elicited by transcranial magnetic stimulation, may be informative for patients with severe early upper-limb impairment.^27–29^ Neuroimaging markers measured within the first 24 hours after stroke, such as collateral status,^30^ reperfusion grading scores,^31,32^ and metrics reflecting lesion growth in large vessel occlusions,^33^ may also add value. Even so, clinical scores are likely to remain more practical for routine clinical use.

A few other considerations qualify our results. First, we took a pragmatic approach to identify a set of bedside predictors. While this approach may not yield the most refined predictor set, it provides a practical solution for clinical settings. Figure 1 further indicates that only limited gains in accuracy can be expected by adding more clinical motor tests as predictors. Second, XGBoost does not account for within-patient measurement correlations, so short-term fluctuations in early motor scores may not be fully captured by the model. Third, patients with hemorrhagic and recurrent stroke were excluded, but their recovery trajectories differ from those of ischemic stroke.^34^ Fourth, we chose the ARAT as the target for prediction, which is a unilateral upper-limb capacity measure. Other clinically relevant aspects of functional performance, such as real-world or bimanual arm use, may require different outcome measures and additional predictors and were beyond the scope of this study.

Machine learning holds promise for predicting upper-limb outcome after stroke, but more complex models are not necessarily better. Tree-based methods, such as XGBoost, can capture individual variation in recovery more effectively than linear models,^12,21^ but increased model complexity can reduce interpretability and transparency and should therefore be justified. For example, temporal models such as recurrent neural networks may better capture recovery trajectories, while multimodal architectures could integrate inputs from different stages of care. This would require linking currently fragmented data sources across the stroke care pathway.

In conclusion, we present a machine learning model that accurately predicts upper-limb outcome after stroke within the first 72 hours using bedside clinical tests. Its early applicability holds promise for integration into hospital stroke units, where it could support clinicians in setting realistic treatment goals, informing patients, and improving discharge planning. However, for optimal clinical utility, prediction performance for patients with severe early impairment needs to be improved. Beyond that, further steps are required before clinical implementation, including external validation, feasibility evaluation in routine care, integration of the model into digital workflows, and assessment of the impact of model use on rehabilitation processes and outcomes.

## ARTICLE INFORMATION

### Sources of Funding

### Disclosures

None.

### Supplemental Material

Table S1

Figures S1–S4

TRIPOD-AI Checklist

## Supplementary Material

## Appendix

### The ICAI Stroke Lab Collaborators

Daniël Bos, Sandra Cornelissen, Ellen Hu, Peter van Hulst, Xi Li, Hester Lingsma, Frank te Nijenhuis, Bob Roozenbeek, Danny Ruijters, Milou Silkens, Ruisheng Su, Sandra Sülz, and Theo van Walsum. The collaborators provided valuable feedback on the study design, analysis methodology, and interpretation of the results.
