# Supplementary material for: Accuracy of Machine Learning to Predict Upper-Limb Outcome Within the First 72 Hours Poststroke
Source: Stroke. 2026 Jun 10;57(8):2493–9. doi: 10.1161/STROKEAHA.125.054989 (PMC13399730; doi:10.1161/STROKEAHA.125.054989)
Supplement: Supplementary file 1 [file str-57-2493-s001.pdf]

Supplemental Material

The methods for fitting the XGBoost model, selecting the predictor set, and evaluating the model are shown in Figure S1.

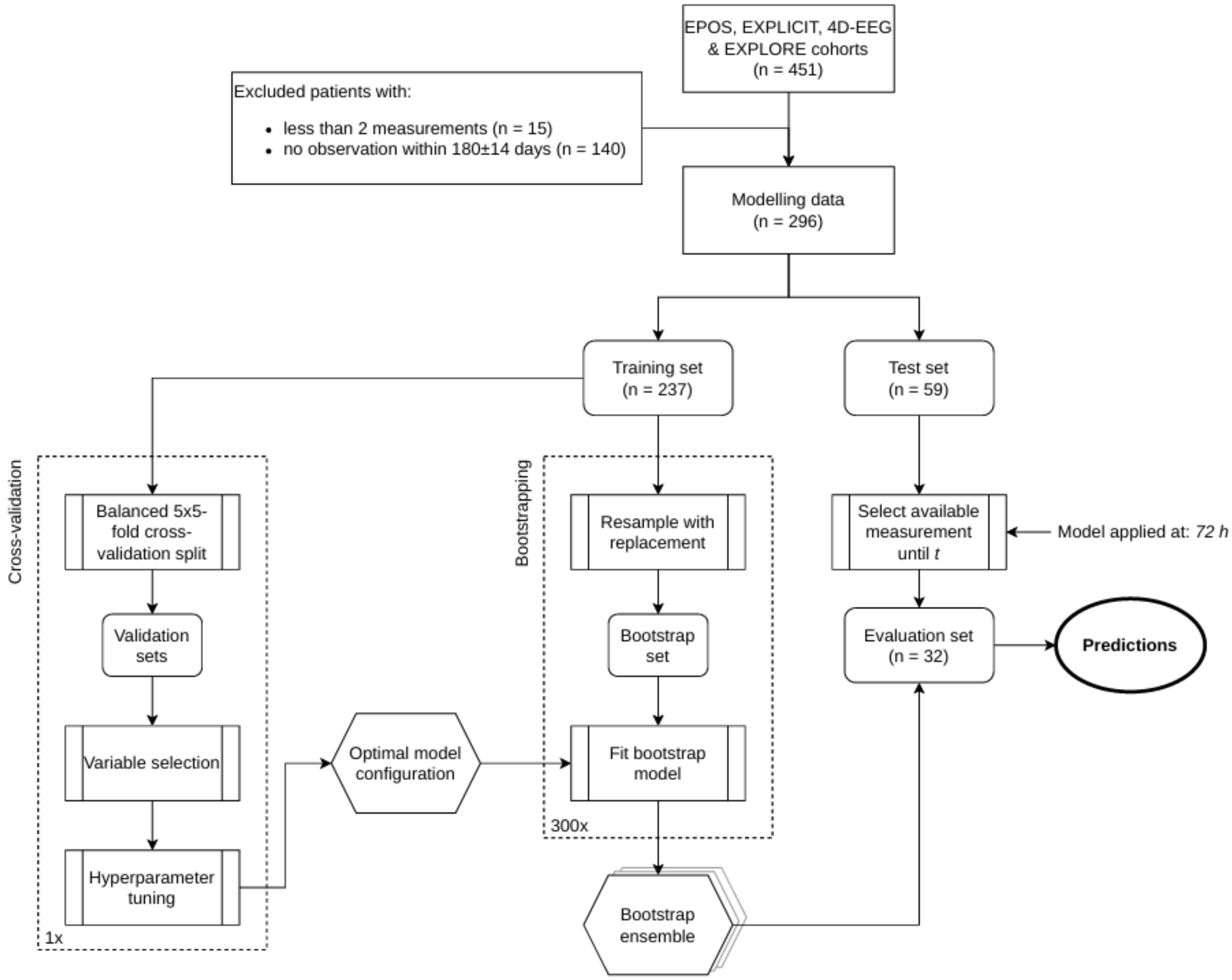

Figure S1 Flow chart depicting the data inclusion, variable selection, and model-building process. Bootstrapping was employed to generate an ensemble of models evaluated on a random selection of patients from a hold-out set (n=59). All models in the ensemble shared the same configuration, optimised through a 5-fold, 5-repeat cross-validation procedure.

Supplementary Figure S2 shows the distribution of first assessment timing for all 296 included patients. As reported in the main text, 54% (161/296) had their baseline measurement taken within 72 hours of stroke onset. The remaining 46% were assessed later, with a median of 3 days (IQR = [2, 7]) across the full sample.

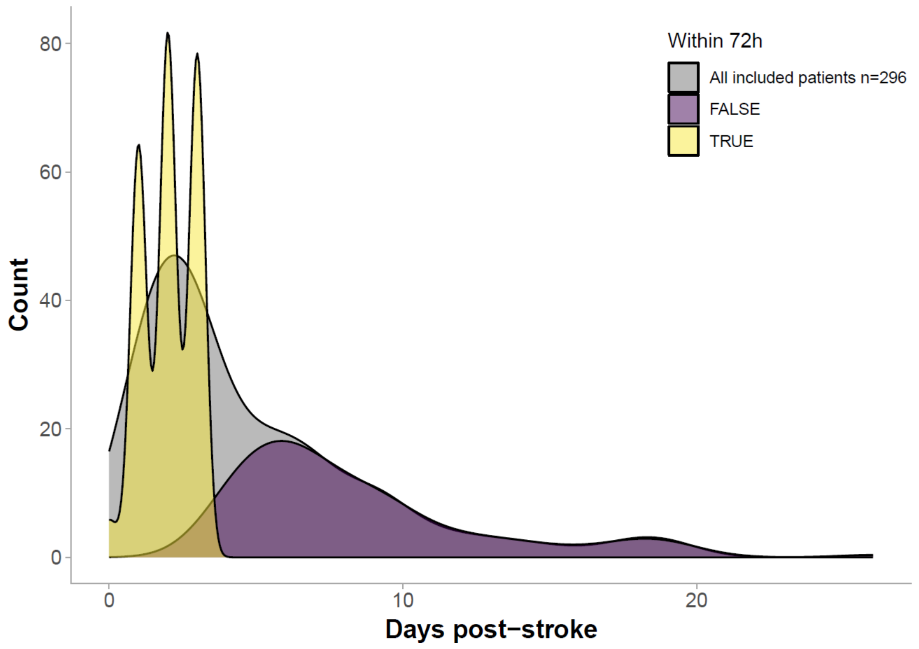

Figure S2 Distribution of first assessment timing in days relative to stroke onset for all included patients (grey), stratified by whether the assessment occurred within 72 hours (yellow) or after 72 hours (purple).

Figure S3 presents three examples of six-month ARAT predictions, along with their corresponding probability density distributions and 80% prediction intervals, for three representative patients. The left column (Patient 1) is an example from the largest subgroup of patients who exhibit some baseline motor function and/or low stroke severity, typically achieving favorable recovery at six months. The middle column (Patient 2) is an example of a patient with unfavorable outcomes, often characterized by low baseline motor scores and high stroke severity. The right column (Patient 3) demonstrates a challenging example of a patient with higher prediction errors. These patients frequently display unexpected recovery patterns, such as severe baseline impairments followed by sudden recovery or mild baseline impairments with no significant upper-limb recovery.

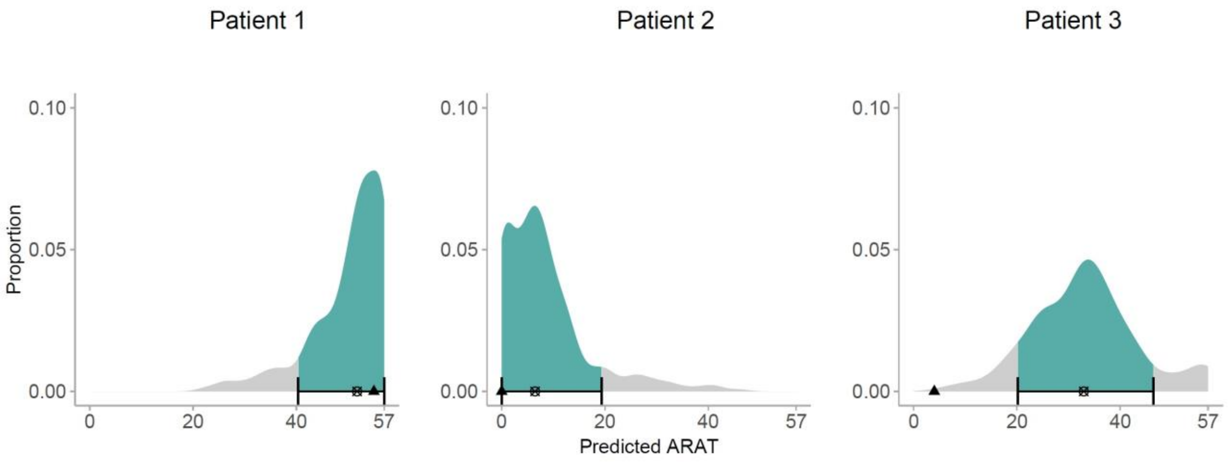

Day	MISA	VFE	FMUE	NIHSS
3	19	partial	36	5

Day	MISA	VFE	FMUE	NIHSS
1	0	none	0	17

Day	MISA	VFE	FMUE	NIHSS
3	9	none	9	9

Baseline predictors

Figure S3 Individual probability density distributions (grey plus green) of six-month ARAT predictions and corresponding 80% prediction intervals (green only) generated by the final bedside model for three representative patients within 72 hours post-stroke. Prediction intervals are shown as error bars with dark-shaded areas in the distributions. Also shown is the median predicted ARAT (⨂) and the observed outcomes at follow-up (▲). Wider prediction intervals and flatter density distributions indicate greater uncertainty in individual predictions. These intervals, therefore, provide clinicians with valuable information about the reliability of each prediction. MISA: Shoulder Abduction from Motricity Index, VFE: Voluntary Finger Extension from the Fugl-Meyer Upper Extremity, ARAT: Action Research Arm Test, FM-UE: Fugl-Meyer Upper-Extremity, NIHSS: National Institutes of Health Stroke Scale.

Figure S4 shows model prediction performance stratified by three baseline FM-UE severity groups, defined at the first assessment within 72 hours post-stroke. While prediction accuracy is generally stable in patients with mild to moderate early impairment, it is more variable in patients with severe early paresis (FM-UE 0–18). In this group, a small number of patients (n=5) achieve substantially better outcomes than predicted, whereas most show limited recovery. Owing to the limited sample size within each severity stratum, these results are intended to illustrate qualitative differences in model performance rather than to support formal subgroup comparisons

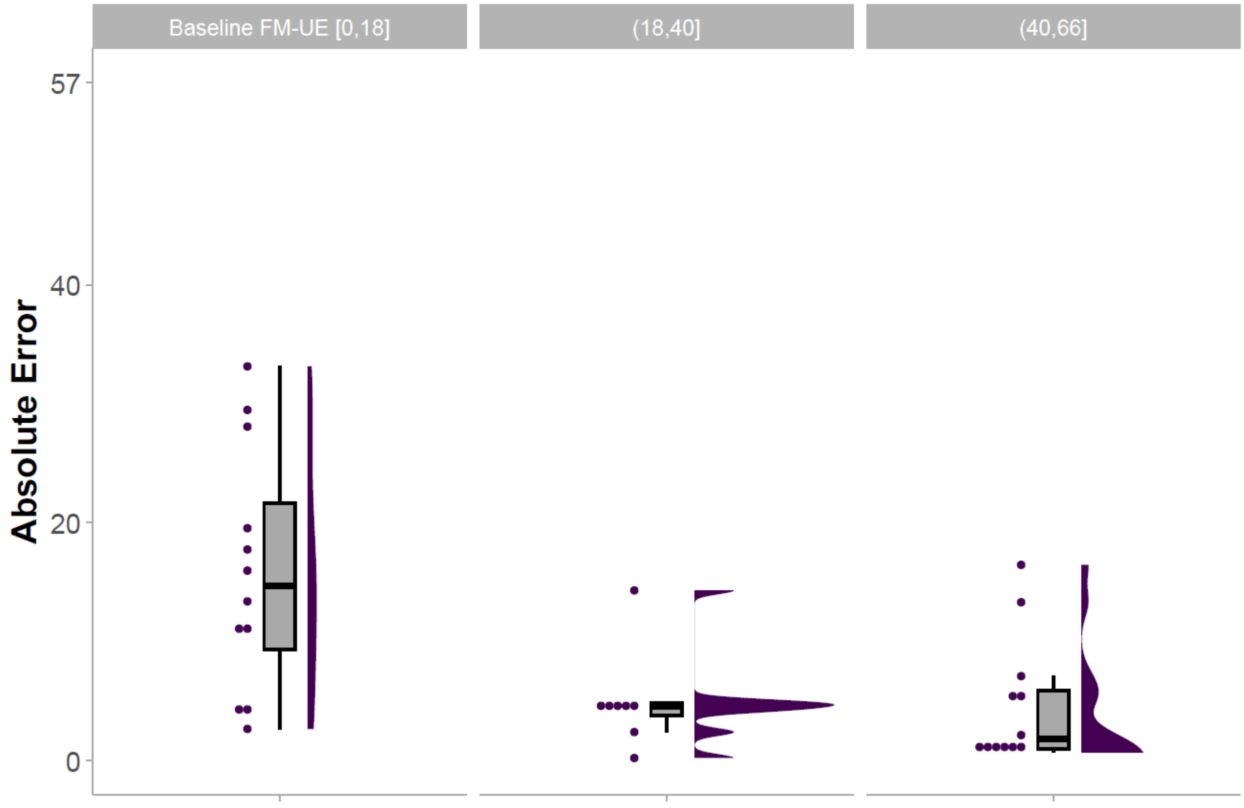

Figure S4 Six-month ARAT prediction errors for the model for patients from the hold-out set with a measurement within 72 hours post-stroke, stratified by their baseline FM-UE score (0–18, 19–40, and 41–66). Boxplots depict the median, interquartile range (IQR), lower whisker (Q1 − 1.5 × IQR), and upper whisker (Q3 + 1.5 × IQR), with outliers indicated as points outside this range. Individual prediction errors are represented as dots, and the lateral density plots illustrate the distribution of errors within each stratum.FM-UE: Fugl-Meyer Upper-Extremity, ARAT: Action Research Arm Test.

Among the 17 baseline variables compared between patients included in the analysis and those excluded due to unavailable outcome measurement, most showed good balance, with absolute standardized mean differences (ASMDs) below 0.2. Three variables exceeded the threshold for a meaningful difference. The total NIHSS score and NIHSS item 11 (extinction and inattention) were both at the threshold (ASMD = 0.20), indicating modest differences in baseline stroke severity. In addition, the Bamford classification showed a somewhat larger imbalance (ASMD = 0.27). All other variables demonstrated negligible differences between the two groups.

Table S1 Baseline characteristics of patients included and excluded from the analysis and their Absolute Standardized Mean Difference (ASMD). Baseline was defined as the first available measurement for each patient (Mean = 5 days, SD = 4.6 days).

	**Analysis sample** Mean (SD) or n (%) n = 296	**Excluded patients** Mean (SD) or n (%) n = 155	**ASMD**

**Age [years]**	65 (13)	65 (15)	0.04
**Sex [females]**	138 (47%)	80 (52%)	0.14
**Type of stroke (Bamford classification)**			0.27
Lacunar Cerebral Infarct (LACI)	151 (51%)	68 (44%)	
Partial Anterior Circulation Infarct (PACI)	101 (34%)	50 (32%)	
Total Anterior Circulation Infarct (TACI)	44 (15%)	37 (24%)	
**Treatment with recombinant tissue plasminogen activator**			0.01
Yes	68 (23%)	36 (23%)	
No	227 (77%)	119 (77%)	
Missing	1 (0.3%)	0	
**Affect bodyside (right)**	111 (38%)	66 (43%)	0.15
**Dominant hand (right)**	272 (92%)	144 (93%)	0.15
**National Institutes of Health Stroke Scale (NIHSS) [0-42]**	8 (5)	9 (6)	0.20
**Baseline Action Research Arm Test (ARAT) [0-57]**	14 (19)	13 (20)	0.07
**Baseline Shoulder Abduction of Motricity Index**			0.13
0: No voluntary movement	96 (32%)	53 (34%)	
9: Voluntary activity palpable, no movement visible	28 (10%)	16 (10%)	
14: Voluntary movement seeable but not seeable in total movement range	62 (22%)	37 (24%)	
19: Voluntary movement across total movement range, not possible against resistance	15 (5%)	8 (5%)	
25: Voluntary movement against resistance, but weaker than contralateral side	65 (22%)	28 (18%)	
33: Normal strength in comparison with contralateral side	28 (10%)	13 (8%)	
**Baseline Finger Extension of Fugl-Meyer Assessment**			0.17
None	153 (52%)	90 (58%)	
Partial	62 (21%)	28 (18%)	

Full	81 (27%)	37 (24%)	
**Baseline Fugl-Meyer Upper Extremity [0-66]**	25 (22)	24 (23)	0.06
**Motricity Index Arm [0-100]**	39 (34)	36 (34)	0.10
**Motricity Index Leg [0-100]**	50 (33)	48 (33)	0.04
**NIHSS 8 - Sensation**			0.16
No sensory loss	135 (46%)	68 (43%)	
Mild to moderate sensory loss	126 (43%)	61 (39%)	
Severe or total sensory loss	35 (12%)	26 (17%)	
**NIHSS 11 – Extinction and inattention (neglect)**			0.20
No abnormality	195 (66%)	88 (57%)	
Visual, tactile, auditory, spatial or personal inattention	45 (15%)	30 (19%)	
Profound hemi-inattention or extinction to more than one modality	56 (19%)	37 (24%)	
